# Association between changes in habitual stepping activity and cognition in older adults

**DOI:** 10.1038/s41598-024-58833-x

**Published:** 2024-04-05

**Authors:** Myles W. O’Brien, Nick W. Bray, Isadora Quirion, Shirko Ahmadi, Pierre Faivre, Francois Gallant, Caroline Gagnon, Martin Sénéchal, Olivier Dupuy, Mathieu Bélanger, Said Mekari

**Affiliations:** 1 Department of Medicine, Université de Sherbrooke, Sherbrooke, Québec Canada; 2 Centre de Formation Médicale du Nouveau-Brunswick, Université de Sherbrooke, Moncton, Canada; 3 Recovery and Performance Laboratory, Faculty of Medicine, Memorial University of Newfoundland, St. John’s, Newfoundland and Labrador Canada; 4 Laboratory MOVE (EA 6314), Faculty of Sport Sciences, University of Poitiers, Poitiers, France; 5 Department of Family Medicine, Dalhousie University, Halifax, NS Canada; 6 Vitalité Health Network, Moncton, Canada; 7 Faculty of Kinesiology, University of New Brunswick, New Brunswick, Canada; 8 Cardiometabolic Exercise & Lifestyle Laboratory, Faculty of Kinesiology, University of New Brunswick, New Brunswick, Canada

**Keywords:** Executive function, Processing speed, Average-real variability, Fitbit intervention, Personalized health, Community interventions, Ageing, Medical research

## Abstract

Advancing age is associated with declines in cognitive function. Although physical activity is thought to protect against this decline, it is unclear how a short-term uptake in daily steps or a decline in day-to-day step variability may contribute to cognition among older adults. We tested associations between changes in step counts, day-to-day step variability and executive cognitive functions among older adults taking part in a physical activity intervention. Thirty-seven older adults (33 females; 71.4 ± 6.3 years) completed a 10-week personalized physical activity intervention. Participants wore a Fitbit to measure daily step counts throughout the study. They also completed a computerized Stroop task before and after the intervention. Average step counts and step count variability via average-real-variability (ARV) were determined. Compared to pre-intervention, step counts increased (*p* < 0.001) and step variability decreased post-intervention (*p* = 0.04). Models describing the changes in step counts and ARV over the 10-weeks were cubic (both, *p* < 0.04). Reaction times during the simple (*p* = 0.002) and switching (*p* = 0.04) conditions were faster post-intervention. Change in step variability was positively associated with the change in reaction time for the switching condition (β = 0.029, *p* = 0.002). On average, a reduction in day-to-day step variability was associated with improvements in cognitive flexibility.

## Introduction

The cognitive decline that commonly occurs with aging is characterized by a decrease in cognitive flexibility as noted by less efficient executive functions^1^. Impaired executive functions, particularly related to inhibitory control, marks the early stages of numerous neuropsychological conditions, such as Alzheimer’s disease^2,3^. Regular physical activity can attenuate the lose of inhibitory control that is associated with aging or even improve cognitive functions among older adults^4–6^. Given the growing challenges associated with aging in western society, the identification of strategies that preserve executive functions to minimize the burden associated with aging-related cognitive declines are required. Specifically, a better understanding of the characteristics of physical activity most likely to promote better executive functions among older adults is needed to guide future population health strategies.

Intervention studies equating physical activity with better cognitive health have primarily focused on structured physical activity or exercise training in controlled environments^4,6–8^. The impact of habitual activity on cognitive functions is an important aspect of active aging and strongly indicates that more free-living activity is associated with better cognition. Older females who were more physically active as assessed via accelerometry exhibited faster reaction times during the executive function portion of the Trail Making Task than their less active peers^9^. Further, we have previously documented that higher habitual physical activity levels are cross-sectionally associated with faster reaction times and intra-task oxygenation of the prefrontal cortex on the Switching conditions (i.e., the most demanding of executive function) of a colour-word Stroop task^10^. Best et al.^11^ demonstrated that older females who improved their executive function following training showed better adherence to physical activity as assessed via the Physical Activity Scale for the Elderly post-training. Older adults’ real life daily physical activity levels tend to exhibit considerable variability. While regular physical activity is associated with better cognition, the relationship of between-individual variations in change in physical activity because of an activity intervention on changes in cognition are unclear.

In addition to average measures of activity each day, there is evidence suggesting that the day-to-day pattern by which activity is accumulated could have a physiological impact^12,13^. For example, individuals accumulating the same number of steps each week but over one-two days may exhibit divergent physiological responses and possibly greater health effects to those who spread it out consistently over multiple days^14^. While persons who consistently accumulated > 8000 steps/day and those who accumulated > 8000 over one-two days per week exhibit similar, lower risks of all-cause mortality compared to their less active counterpart^15^, the physiological stimulus is inherently different between groups. A more consistent, highly active lifestyle that avoids repeated bouts of low activity could potentially result in a more sustained cardiovascular and neural stimulus that may improve cognitive health, but this is unclear. Individualized interventional strategies that target regular day-to-day physical activity levels may therefore promote better improvements in cognition.

The purpose of this secondary analysis of a single group pre-post study was to investigate whether within-individual changes in step count and in day-to-day step count variability relates to changes in executive function among older adults participating in a 10-week personalized physical activity intervention. We hypothesized that older adults with an uptake in step count and a decline in day-to-day step count variability will present a decline in reaction time associated with the Switching condition of the Stroop task.

## Methods

### Participants

Forty-three older adults living in the community gave informed consent to participate in the present study, however five had missing stepping data across the intervention, resulting in a final sample of *n* = 37. While there is little work to inform a sample size calculation based on analyses of changes in stepping patterns with changes in cognition, a correlational effect size of a moderate level *r* = 0.50, estimated that a minimum of 29 participants were needed assuming a two-tailed, α = 0.05, and power of 80% (G*Power 3.1). Participants were recruited through local community programs (e.g., Rotary club, club d’age d’or, Dames de l’Acadie), social media, and through word of mouth.

To be included, participants had to: be at least 55 years of age, be able to ambulate independently, be cognitively healthy (mini-mental state examination score > 24), have normal-to-corrected vision, not have a history of neurological or psychiatric disorder, color blindness, surgery with general anesthesia in the past six months, involuntary tremors, epilepsy, or drug/alcohol problems. All participants were right-handed and no participants were smokers. One participant was taking medication for depression, one participant was on estrogen therapy, 16 were taking a statin medication, and 14 were taking medication for high blood pressure.

The protocol was reviewed and approved by the Institutional Research Ethics Board at the Université de Sherbrooke and was conducted in accordance with the declaration of Helsinki.

### Physical activity intervention

The 10-weeks intervention was co-developed with older adults from the community and community service providers. It was designed based on the evidence-supported dynamic socio-ecological model of health behaviour change^16^ in which interventions simultaneously address a combination of factors such as personal, social, physical environment and empirical evidence on optimizing cognition in older adults^17^. The program was structured around five, group based aerobic and resistance training activities (i.e., walking club, resistance training sessions with a qualified professional, yoga courses, pickleball, and ice skating). Each week, participants were asked to attend at least three of a possible sixteen group-based sessions (minimum of 150 min of physical activity a week). The group sessions were 50 min and had an exercise intensity of moderate-to-vigorous. Participants were asked to log their physical activity time at the end of each week. Log reports show that every participant completed a minimum of 150 min of moderate-to-vigorous physical activity during the 10 weeks of the study.

### Habitual activity monitoring

Participants were equipped with a FitBit (Fitbit Inspire 2 HR, California, USA) to wear on their wrist for the duration of the study. Participants were instructed to charge the device approximately every 72 h. To be included in the analyses, participants had to have worn the device at least 4 days per week for at least 10 h/day based on detected heart rate and > 500 steps/day (n = 5/43 excluded).

Step counts determined from the Fitbit have been demonstrated to exhibit acceptable validity in older adults living in the community^18^. In addition to total step counts, day-to-day step count variability was calculated using average real-variability (ARV). ARV is a commonly used metric in cardiovascular research that quantifies variability as the absolute difference between subsequent measurement periods (e.g., beat-to-beat, day-to-day, etc.)^19^ thus, accounting for the temporal effects that are otherwise not considered in other variability metrics (e.g., standard deviation).

ARV was calculated by determining the difference between each given day of the week and the previous day (e.g., steps ARV_MondayTuesday_ = absolute [Tuesday steps – Monday steps]). ARV for all days of wear time were then averaged for each week within each participant. ARV reflects the variability between successive days and is determined as the average absolute difference between successive daily habitual activity measurements as in Eq. 1, where *k* is the order of each day and *N* corresponds to the number of days^20^.

1 $$Step Variability: ARV= \frac{1}{N-1}\sum_{k=1}^{N-1}\left|{Steps}_{k+1}-{Steps}_{k}\right|$$

### Anthropometrics, systemic hemodynamics, six-minute walk test

Before and after the intervention, heart rate (via radial pulse), blood pressure (via sphygmomanometer and brachial cuff), and anthropometrics (i.e., weight and height via stadiometer) were determined. Body mass index was calculated as weight ÷ height^2^. Aerobic fitness was assessed via the six-minute walk test (6MWT), which was completed according to standardized procedures provided by the American Thoracic Society^21^. The 6MWT was conducted in an open gym, and the course was marked by red cones placed 30 m apart. The cumulative distance covered over the six minutes was recorded to the nearest centimetre.

### Cognitive tests

Before and after the intervention, participants completed the computerized Stroop task. This version of the Stroop task has been successfully used by our team in previous intervention studies^8,22^. The task which included three different stages (naming, inhibition, and switching) performed sequentially with the right hand and is openly available elsewhere^23^.

They responded using the "u", "i", "o", and "p" keys on a keyboard, corresponding to different fingers and colors: index finger for "u" (red), middle finger for "i" (green), ring finger for "o" (blue), and little finger for "p" (yellow). Each stage began with a set of practice rounds—12 for the first two stages and 20 for the last. Every stage consisted of 60 trials, each starting with a fixation cross for 500 ms, followed by a word display for 3000 ms. Participants had a 5-min rest between stages. Average reaction times were calculated for the 60 trials in each of the three Stroop test stages.

In the first stage, naming, participants identified words (RED, BLUE, YELLOW, GREEN) by pressing the corresponding color-coded key. This stage assessed processing speed. The second stage involved an inhibition task, where participants identified the font color of incongruent color words (e.g., pressing "i" for BLUE written in green). This stage required inhibition of the instinct to read the word. The third stage, switching, was similar to the inhibition task, but with an added complexity: in 15 out of 60 trials, participants responded to the word instead of the font color if a square replaced the fixation cross, testing cognitive flexibility. Among the three different stages, switching condition was the primary outcome of interest in this study. Incorrect responses in practice and actual trials triggered an "Error" message. Reaction times and total errors were recorded, but only reaction times are reported due to the high accuracy rates (over 98% in naming and inhibition, and over 70% in switching)^24^. Participants initially completed the Mini-Mental State Exam^25^, with scores ranging from 25–30 across all participants.

### Statistical analysis

Normality was tested via Shapiro–Wilk, and a visual inspection. Data were analyzed using parametric statistics. All data are presented as means ± standard deviations unless otherwise stated.

Upon visual inspection of the total step counts and step variability across the baseline and 10-week intervention, these data were modelled using non-linear regression analyses, with the highest term that improved the regression model (e.g., quadratic, cubic, etc.) retained based on the significance of the models R^2^ (f) change. Changes in anthropometrics, 6MWT, and step outcomes were compared via paired *t*-tests. To determine whether reaction times changed from pre-intervention to post-intervention, we conducted a time (pre, post-intervention) ANCOVA adjusted for age, sex, mini-mental state examination separately for each condition.

As outlined in our purpose statement, the primary objective was to examine whether changes in an activity measure were associated with changes in cognition. This was achieved via regression analyses that examined whether changes in step counts or step variability were associated with changes in reaction time for each Stroop condition adjusted for age, sex, mini-mental state examination, and pre-intervention reaction time. Non-linear regressions were first investigated for all primary comparisons, but the incorporation of a higher order term did not improve the predictive capabilities of the model (all*, p* > 0.10). All statistics were completed in SPSS Version 28.0 (IBM, NY). Statistical significance was accepted as *p* < 0.05.

## Results

Participant characteristics are presented in Table 1. The participants included primarily females (n = 33/37) and were cognitively healthy with all MMSE scores > 24 (Table 1). On average, body mass and body mass index were lower, and 6MWT distance was longer following the intervention (all, *p* < 0.001; Table 1).

**Table 1:** Participant characteristics and anthropometrics.

Variable	Participants (n = 37)	
	Pre-Intervention	Post-Intervention
Age (years)	71.4 ± 6.3	
Sex (males, females)	4, 33	
MMSE^a^ (out of 30)	28.9 ± 1.3	
Height (cm)	159.7 ± 8.0	
Body mass (kg)	78.8 ± 13.6	77.7 ± 13.8*
Body mass index (kg/m^2^)	31.0 ± 5.4	30.6 ± 5.5*
6MWT (m)	532 ± 112	577 ± 74*
Resting heart rate (beats/min)	68 ± 11	69 ± 11
Systolic blood pressure (mmHg)	135 ± 15	135 ± 13
Diastolic blood pressure (mmHg)	79 ± 8	78 ± 9

Data presented as means ± standard deviation or proportion. 6MWT, six-minute walk test. The impact of the intervention on body mass and body mass index were compared pre-post via paired *t*-tests.

*p < 0.05 to pre-intervention.

^a^n = 36 for mini-mental state examination (MMSE).

**Figure 1:**
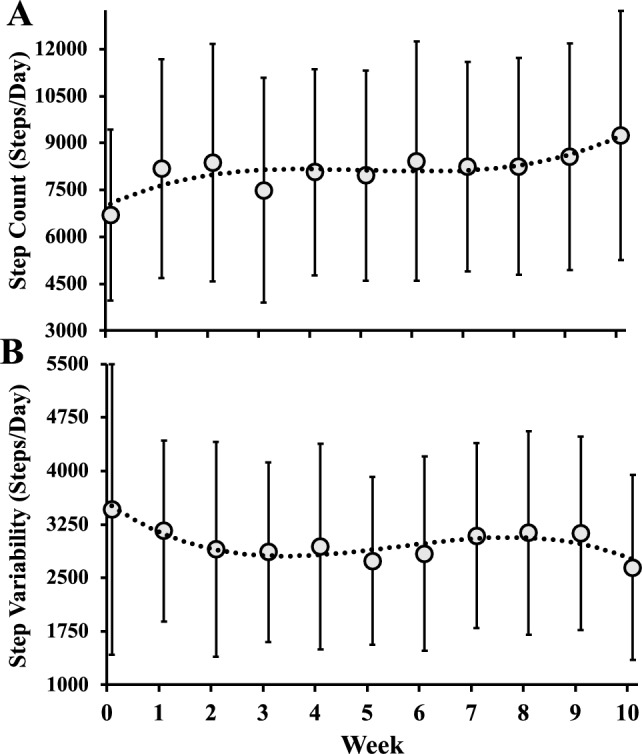
Changes in daily step count (**A**) and day-to-day step-variability as assessed via average-real-variability (**B**) each week from baseline (week 0) to the end of the 10-week intervention. Data presented as means ± SD. The relationships in (**A**,**B**) were best characterized by a cubic function. n = 37.

The step counts and day-to-day step variability before and during the 10-week intervention are presented in Fig. 1. Total step counts and step count variability over the 10-weeks were best characterized using a cubic function with steeper changes at the beginning and end of the intervention, with the intervention increasing step counts (Fig. 1A; model: *p* = 0.01) and decreasing the day-to-day variability (Fig. 1B; model: *p* = 0.04). Specifically, models incorporating a cubic term resulted in a statistically better model fit than the linear or quadratic model for total steps and step count variability (both, *p* < 0.05), but a quadratic term did not further improve the models (both, *p* > 0.10). Comparing pre-intervention to the last week of intervention, step counts increased (*p* < 0.001) and step variability decreased (ARV; *p* = 0.04).

**Figure 2:**
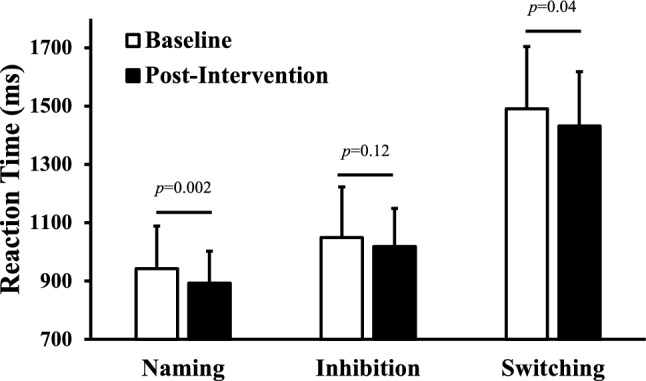
The change in Stroop task reaction time at baseline (white bars) and after the 10-week physical activity intervention (black bars) for the naming, inhibition, and switching condition. Data were compared via a repeated measures ANOVA adjusted for age, sex, and mini-mental state examination, with Bonferroni post-hoc testing with within-condition differences presented. Data presented as means ± SD. Between condition differences in reaction time were observed (naming < inhibition < switching). n = 37.

Following the intervention, reaction time during the simple naming condition was faster (*p* = 0.002; Fig. 2A), but the inhibition condition was not different pre-post (*p* = 0.12; Fig. 2B). The switching condition, which represents the most difficult task tested was completed faster post-intervention (*p* = 0.04; Fig. 2C).

**Figure 3:**
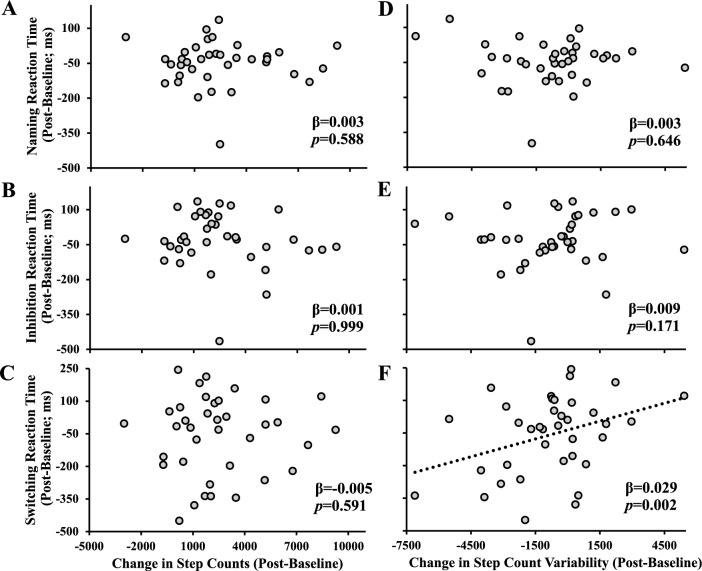
The relationship between the change (post-intervention minus pre-intervention) in step counts and change in step variability with the change in reaction time for the Naming (**A**,**D**), Inhibition (**B**,**E**), and Switching condition (**C**,**F**). Linear regressions were conducted between the change in each step outcome with the change in each condition’s reaction time adjusted for age, sex, mini-mental state examination, and pre-intervention reaction time. n = 37.

Changes in total step counts from post–pre intervention was not associated with the change in reaction time for the naming (*p* = 0.59; Fig. 3A), inhibition (*p* = 0.99; Fig. 3B), or switching condition (*p* = 0.59; Fig. 3C). Changes in day-to-day step variability were not associated with the change in reaction time for naming (*p* = 0.65; Fig. 3D) and inhibition (*p* = 0.17; Fig. 3E), but were positively associated with the change in reaction time for the switching condition (*p* = 0.002; Fig. 3F). Specifically, improvements in step count variability (lower variability) were associated with greater improvements in switching reaction time.

## Discussion

Consistent with our hypothesis, a change toward becoming more consistent in day-to-day step variability, but not toward more total steps, was associated with faster Switching reaction times. Accordingly, this study suggests that the promotion of more consistent day-day participation in physical activity among older adults may be a promising intervention target to sustain better executive function.

The Stroop task is a well-established neuropsychological assessment of simple and executive functions^26^, with the switching task being the most sensitive to aging^27^. Related, the time required for the Switching task is ~ 1.5 times longer than the simple naming condition (Fig. 2), and reaction time during this more cognitively challenging task was improved following the intervention. A faster reaction time reflects better cognitive flexibility that may translate into a meaningfully reduced risk of developing cognitive disorders, given that impairments in executive function are among the initial characteristics of cognitive decline^3^. This study adds to the literature by demonstrating that changes in step counts variability were positively related to improvements in cognitive flexibility. While national physical activity recommendations for older adults focus on physical activity for the whole week^28^, our data indicate that a lifestyle with less day-to-day variability and ideally a regularly high step counts might be advantageous for brain health in older adults living in community. Altogether, the personalized physical activity intervention from which these data originated was associated with an increase in average step count, improvement in step variability, and better cognitive flexibility. Whereas it needs to be tested within an experimental study, the intervention model implemented may be used as a guide to help older adults achieve lower variability in daily physical activity.

Despite results from the current analysis, more work is required to establish the clinical relevance of physical activity variability. On average, we noted that reducing the subsequent days steps to within ± 2500 steps of the prior day from ± 3500 steps/day was associated with improvements in cognitive flexibility. A reduction in ARV might provoke other health benefits which need to be investigated. While the mechanisms underpinning the cognitive benefits of transitioning from a yo-yoing day-to-day stepping patterns to maintaining a similar number of steps each day are not determined, it is possible that reducing the number of days of lower step counts contributes to better cerebrovascular function^29^, prefrontal cortex tissue perfusion^10^, and/or other mechanisms (e.g., brain-derived neurotropic factor, myokine release, insulin growth factor 1^30^ etc.). Neuroimaging studies have documented that the improvement in cognition following exercise training may be due to greater prefrontal cortex oxygenation^31,24^, which may be a result of better endothelial-derived nitric oxide production and thus vasodilation of cerebral vessels and/or possibly through exercise-induced angiogenesis (i.e., creation of new capillaries) as evident in animal models^32^, which would enhance cerebral perfusion and possibly improve cognition. Although this study was not designed to test mechanisms, it is plausible that any individual or combination of these factors are responsible for the cognitive improvements observed in combination with the decline of ARV. Given that the participants also increased their step counts, it is plausible that the combination of more activity and a lower variability were responsible for the improvements in Switching reaction time, rather than the lower variability describing those who are consistently inactive. However, the independent effects of these stepping phenotypes (average versus variability) cannot be discerned and larger sample sizes that investigate the combination of high-low step counts/variability are warranted.

While interpreting our results, our findings may not be generalizable to older adults with mild or severe cognitive impairments, especially those who have difficulty exercising independently. Given the over-representation of women in the current study, it is also possible that results are more applicable to females. While the inclusion of an objective monitor is a strength of the study, it is primarily validated for step counts^18^ and does not provide valid measures of physical activity intensity or habitual posture that could influence cognition outcomes^10^. However, step counts are highly translatable to the general population. The objective of the study was to examine the relationship between changes in stepping patterns with changes in Stroop performance, rather than the effectiveness of the intervention per se which would benefit from a control group to rule out any practice effects and isolate the impact of the activity intervention specifically. The information gained from this study provides useful insight into the development of strategies that could help older adults lead more active, and consistently active lifestyles. However, future research should consider the follow-up of participants not exposed to a physical activity intervention to observe whether associated noted in the current study can be replicated in other contexts.

## Conclusion

This study highlights that beyond the already recognized benefits of overall physical activity, it may become relevant to promote lowering day-to-day variability in physical activity as a strategy to improve executive function of older adults. As suggested by this study, a community-based personalized physical activity program may help achieve higher and more consistent step counts as well as lead to better executive function among older adults.

## Data Availability

The datasets generated during and/or analyzed during the current study are not publicly available, but are available from the corresponding author via email upon reasonable request.
